# Arthroscopic suture retrievers and shuttles: a biomechanical investigation of the force required for tendon penetration and defect size

**DOI:** 10.1186/s12891-015-0794-9

**Published:** 2015-11-17

**Authors:** Christopher G. Lenz, Karl Wieser, Georg Lajtai, Dominik C. Meyer

**Affiliations:** Orthopaedic Department, Balgrist Hospital, University of Zurich, Forchstrasse 340, CH-8008 Zurich, Switzerland; Orthopaedic Department, Private Hospital Maria Hilf, Klagenfurt, Austria

**Keywords:** Rotator cuff, Tendon, Suture retriever, Suture shuttle, Lesion size, Iatrogenic damage

## Abstract

**Background:**

To compare instruments designed for arthroscopic suture handling during arthroscopic rotator cuff repair, to assess the force needed to penetrate the tendon, and to evaluate the residual defect size.

**Methods:**

Twenty-one instruments were each tested ten times on thawed sheep infraspinatus tendons. The force needed to pierce the tendon with each instrument was measured using a custom setup. Bone wax plates were used to make the perforation marks visible and to quantify the lesions each instrument created.

**Results:**

The force to pierce a tendon had a range of 5.6–18.5 N/mm. Within the group of suture retrievers, the angled instruments required in average 85 % higher forces than straight instruments. The lesion area had a range of 2–7 mm^2^. Suture retrievers produced significantly larger lesion sizes compared with suture shuttles.

**Conclusion:**

For the identical task of passing a suture through a tendon, differences exist regarding the ease of tendon penetration and potential damage to the tendon for different tools. The design, function, and resulting lesion size may be relevant and important for surgical handling and to avoid excess structural damage to the tendon. These results suggest that choosing the most appropriate tools for arthroscopic suture stitching influences the ease of handling and final integrity of the tissue.

**Level of evidence:**

Mechanical evaluation of surgical devices.

**Electronic supplementary material:**

The online version of this article (doi:10.1186/s12891-015-0794-9) contains supplementary material, which is available to authorized users.

## Background

Arthroscopy is the most frequently used approach for the treatment of rotator cuff tears [1] and is a well-established technique for reconstruction of the labroligamentous complex of the shoulder and hip. It is widely accepted that several factors such as genetic predisposition, extrinsic impingement, intrinsic degeneration of tendon tissue and biomechanical aspects of surrounding structures can lead to tears of the rotator cuff, but the pathogenesis still is not fully understood [2]. The use of sutures and suture anchors in rotator cuff repair is a straight-forward and well accepted method, over all associated with a low complication rate [3]. During the past few years, numerous instruments and tools have been designed by different manufacturers for improved handling and to expand the indications for reconstructive arthroscopic surgery. These instruments play an essential role in the success of the surgeries and thus presumably of their corresponding clinical outcomes.

Most of these tools can be classified as either suture retrievers or suture shuttles. A suture retriever typically has a mouth that can be opened and a pointed tip to pierce a tendon, and is suitable to grasp and for pulling or sometimes pushing a suture through the tissue, such as during a rotator cuff repair. A suture shuttle often resembles a long, hollow needle with no mouth opening, and is used to pass a suture, usually monofilamentous, through the device after piercing the tendon. A shuttled suture may then be used for further suturing or to pull in another, definitive suture at a later stage.

Based on our observation that certain instruments require higher forces for tendon perforation leading to considerable defects within the tendon or labral complex, we conducted this biomechanical investigation. We hypothesized that suture shuttles require less force to pierce a tendon and cause less tendon damage than suture retrievers because of their slimmer design, though their use potentially increases the number of surgical steps. Recent reports have raised concerns about iatrogenically provoked tendon substance failure medial to the tendon to bone insertion, and any damage caused by the stitch itself is of potentially serious concern [4, 5]. Therefore, we also determined the size and pattern of the lesions that are generated by these instruments.

## Methods

Tools from many of the major manufacturers of suture retrievers and shuttles were included in this study. Twenty-one new instruments were included in this study and photographically documented (Camera: D300s Digital Camera; Nikon Corporation, Tokyo, Japan; Lens: EX Macro 105 mm 1:2.8D; Sigma Corporation of America, Ronkonkoma, NY, USA).

Instruments were categorized as straight and angled suture retrievers (*n* = 12) or suture shuttles (*n* = 9, Additional file 1: Table S1). A gauge was used to measure the diameter of the portion of the instrument that passes through the tissue (Additional file 1: Table S1 is added as additional file providing an overview of used intruments and results). The animals were acquired from a local butchery (Metzgerei Angst AG, Herdernstrasse 61, 8004 Zurich, Switzerland), which is certified according to FSSC 22000 which is approved by the Global Food Safety Initiative (GFSI).

### Force measurement

**Fig. 1:**
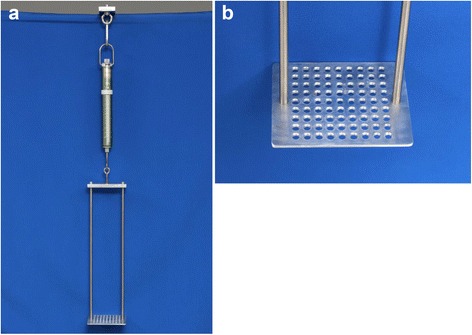
Construction for measurement of applicated force (**a**) Spring scale (**b**) Tray for placement of tendons and bonewax plates

Twenty-one fresh frozen sheep infraspinatus tendons were isolated (mean length: 51 mm, range: 40–61 mm; mean width: 18 mm, range: 11–21 mm). The specimens were stored frozen at −20 °C. Before mechanical testing, the tendons were thawed at room temperature for 12 h. The specimens were kept moist with 0.9 % saline during mechanical testing. A custom testing device was constructed for this study. A sieve-like plateau (10 cm × 12 cm) was created. The tendons were placed on the holes of the plateau, which were carefully chosen and created with sufficient size (6-mm diameter each) to avoid contact between the instruments and the plateau (Fig. 1). The plateau was coupled to a hanging spring scale (Fig. 1). The tendons were placed on the plateau and pierced while ensuring that the force vector from each instrument, based on design, was perpendicular to the spring scale.

**Fig. 2:**
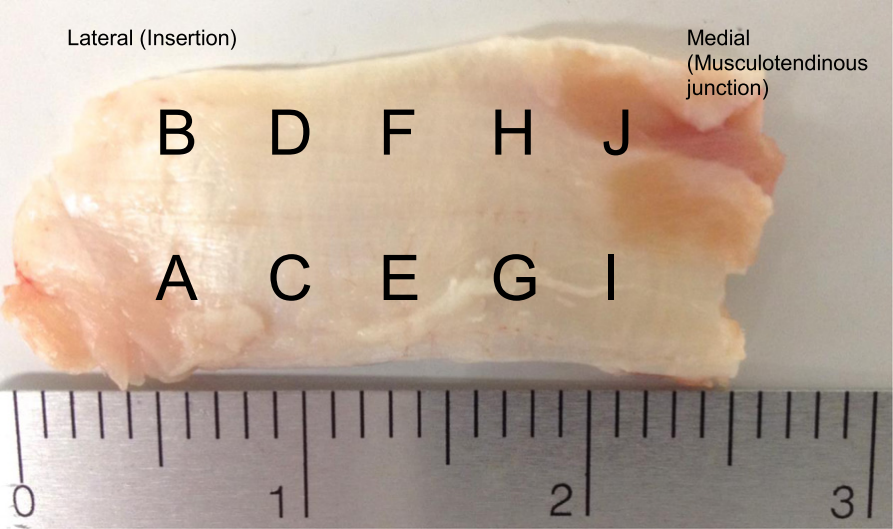
Infraspinatus tendon of the sheep, ten spots were identified and each instrument was used to pierce each spot on the tendon leaving at least 2 mm of space in between

Each instrument was tested ten times with altered tendon positions to allow an equal allocation of pierced spots (Fig. 2). The mean thickness of all ten spots was 1.8 mm (range: 0.8–3.7 mm). To avoid interference, we maintained a minimal distance of 5 mm (>2 mm between edges) between the different spots. One single surgeon performed the mechanical tests in an identical manner. We determined the maximal force (N) that was required to pierce the tendon and measured the thickness (mm) of the tendon at the pierced spots. Suture retrievers were tested without suture in the instrument mouth during forward stitching. To compensate for the somewhat higher resistance of thicker tendons, we calculated the force needed per tendon thickness (N/mm, Additional file 1: Table S1 and Table 1). Instrument subgroups of straight and angled suture retrievers and suture shuttles were analyzed.

**Table 1:** All instruments are listed showing the measured Force in N and the obtained diameter in mm

Instrument	Force in N	Diameter in mm
Arthrex™ Penetrator Suture Retriever II® Straight	8	2.3
Arthrex™ Rhino® Straight Tip	10	2.3
Smith & Nephews™ Arthropierce® Straight	19	2.8
Tornier™ Penetrating Grasper® Straight	15	2.7
Arthrex™ Penetrator Suture Retriever II® 15° Up	34	2.7
Biomet™ Arthropasser® 35° Up	20	2.0
Biomet™ Arthropasser® 45° Left	14	2.5
Smith & Nephews™ ArthroPierce® 35° Up	14	2.8
Smith & Nephews™ ArthroPierce® 45° Right	28	3.1
Tornier™ Birdbeak® 35° Up	23	2.3
Tornier™ Birdbeak® 45° Right	28	2.6
Tornier™ CleverHook® Right	33	2.8
Arthrex™ Suture Lasso® SD Crescent	20	2.0
Smith & Nephews™ Accu-Pass® Big Curve	16	2.1
Smith & Nephews™ Accu-Pass® Crescent	16	1.8
Arthrex™ Quick Pass Lasso® 90° Curve Straight	20	1.6
Arthrex™ SutureLasso® SD 25° Tight Curve Left	19	2.0
ConMed Linvatec™ Spectrum® Suture Passer 45° Right	17	1.9
ConMed Linvatec™ Spectrum® Suture Passer 60° Right	19	2.2
Smith & Nephews™ Accu-Pass® Suture Shuttle 70°	20	2.0
Smith & Nephews™ Suture Shuttle® Left 45° Curve	19	2.5

### Lesion size

**Fig. 3:**
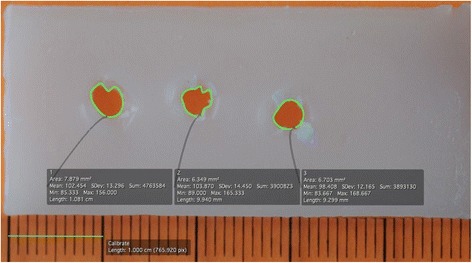
Software to measure the area in mm^2^ of the three perforation marks, which were set with each instruments. Twenty-one bonewax plates were used and sixty-three values could be obtained. Values were compared looking at instrument function and type

Twenty-one bone wax plates (50 × 13 × 3 mm; Ethicon; Johnson & Johnson, New Brunswick, NJ, USA) were used for analysis of lesion size. The plasticity of bone wax plates allows approximation of the pattern of set perforation marks to give an approximation of the tissue damage created by the instruments. Each instrument was used to pierce the same bone wax plate three times (Fig. 3). The diameter, area, and circumference of the set holes were measured using medical imaging software (OsiriX, Advanced Open-Source PACS Workstation DICOM Viewer, Pixmeo SARL, Bernex, Switzerland). In preliminary testing, a setup in which suture retrievers were holding a USP#2 suture in the mouth while guiding through the material, which corresponds to a less common surgical situation, did not show relevant differences. Therefore, the better defined setting of testing each instrument without a suture was used.

### Statistical analysis

Statistical analysis was performed using IBM SPSS Statistics version 20 (SPSS Inc., Chicago, IL, USA). One-way analysis of variance (ANOVA) with instruments as the fixed factor and Bonferroni posthoc tests were used. A two-way nested ANOVA with a fixed factor of design and a random factor of instrument nested into design was also used. Log transformations were applied to improve the normal distribution of the force measurement data. P-values less than 0.05 were considered statistically significant.

## Results

### Force measurement

**Fig. 4:**
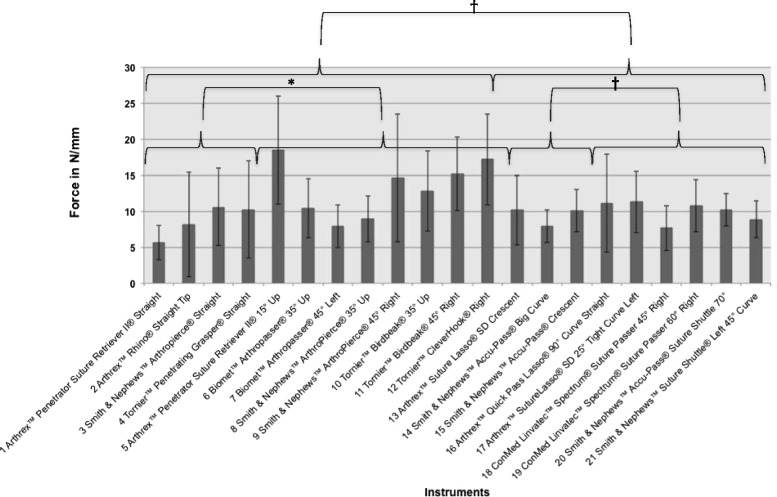
Mean values of all instruments in N/mm are shown. Brackets indicate statistical comparison. Suture Retrievers needed significantly more force to pierce the tendon than Suture Shuttles. The angled Suture Retrievers needed significantly more force to pierce the tendon than straight Suture Retrievers. There was no significant difference of straight and angled instruments within the group of Suture Shuttles. *p = <0.05 † *p*= > 0.05

The force required to pierce the tendons ranged from 5.6 N/mm (Arthrex™ Penetrator Suture Retriever II® Straight) to 18.5 N/mm (Arthrex™ Penetrator Suture Retriever II® 15° Up). The results for each instrument are shown in Additional file 1: Table S1 and Table 1 (in N/mm and N). An overview of the measured force in N/mm of all tested instruments is displayed in Fig. 4. The suture retrievers needed a higher force than the suture shuttles (mean ± standard deviation: 9.8 ± 1.3 versus 11.7 ± 4 N/mm, *p* > 0.05). Within the group of suture retrievers, the straight instruments showed significantly lower force required than the angled instruments (8.7 ± 2.9 versus 13.2 ± 3.9 N/mm, *p* < 0.05). Within the group of suture shuttles, there was no significant difference between the force required for straight and angled instruments (9.4 ± 1.3 versus 10 ± 1.4 N/mm, *p* > 0.05). Overall, straight suture retrievers needed the lowest force (8.7 N/mm) and angled suture retrievers the highest (13.2 N/mm, *p* < 0.05). Considering the diameter of the instruments, there was no indication that larger diameter instruments lead to higher forces required for penetration (Table 2).

**Table 2:** Comparison of diameter and Force in N of the subgroups

	Instrument diameter in mm (mean)	Force in N (mean)
Straight suture retrievers	2.5 ± 0.4	3 ± 4.8
Angled suture retrievers	2.8 ± 0.4	24 ± 7.9
Straight suture shuttles	3.0 ± 0	17 ± 2
Angled suture shuttles	3.6 ± 0.4	19 ± 1.1

### Lesion size

**Fig. 5:**
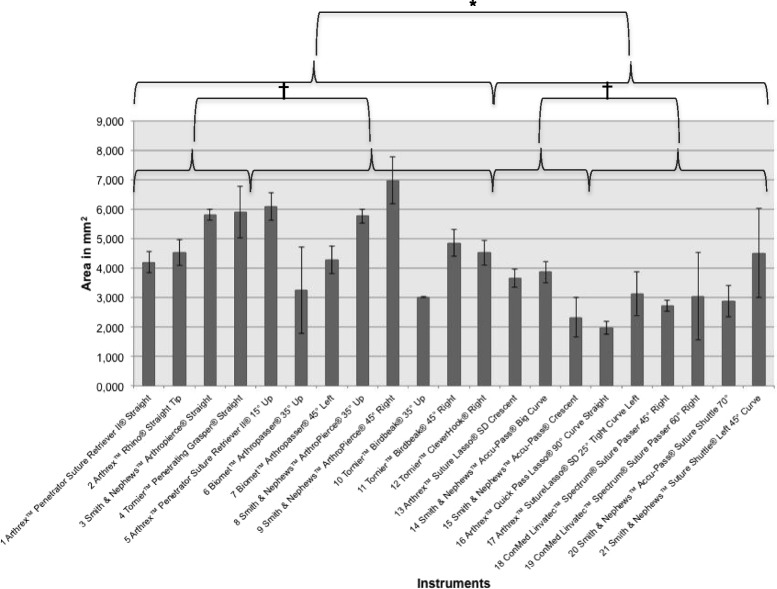
Mean area in mm^2^ of all instruments are shown. Brackets indicate statistical comparison. Suture Retrievers produced a significantly larger area of lesion in mm^2^ of the bone wax plate than Suture Shuttles. Neither, there was a significant difference of straight and angled instruments within the group of Suture Retrievers, nor within the group of Suture Shuttles. *p = <0.05 † *p*= > 0.05

After perforation of the bone wax plates, we measured the area of the lesions created. The results are shown in Table 1. We also measured the diameter. We found a range of lesion areas from 2 mm^2^ (Arthrex™ Quick Pass Lasso® 90° Curve Straight) to 7 mm^2^ (Smith & Nephews™ ArthroPierce® 45° Right). The results from all instruments are shown in Table 1 (in mm) and Fig. 5. The diameters had a range of 1.6–3.1 mm, and the instruments with the lowest and highest diameters also showed the lowest and highest lesion areas (Additional file 1: Table S1).

The suture retrievers produced a significantly larger lesion area compared with the suture shuttles (4.9 ± 1.2 versus 3.1 ± 0.8 mm^2^, *p* < 0.05). Within the suture retrievers, the straight instruments showed a slightly, but not significantly higher lesion size than the angled instruments (5.1 ± 0.9 versus 4.9 ± 1.4 mm^2^, *p* > 0.05). There was also no significant difference between the straight and angled suture shuttles (3.3 ± 0.8 versus 3 ± 0.8 mm^2^, *p* > 0.05). The angled suture shuttles showed the smallest lesion area (3 mm^2^), while straight suture retrievers showed the largest (5.1 mm^2^, *p* < 0.05).

Comparing the designs, straight suture retrievers showed a significantly larger area than straight suture shuttles (5.1 ± 0.9 versus 3.3 ± 0.8 mm^2^, *p* < 0.05). Moreover, angled suture retrievers also showed significantly larger lesion areas than angled suture shuttles (4.9 ± 1.2 versus 3 ± 0.8 mm^2^*p* > 0.05).

No breakage or other kind of damage of the instruments occurred during testing.

## Discussion

Suture retrievers and suture shuttles are indispensable instruments for arthroscopic suture stitching, and various instrument designs are available, including straight and different degrees of angulation in any direction for optimal handling of each specific task. Although the utility of these tools is indisputable, surgeries can require that large numbers of stitches be pierced through small areas of tendon, making smooth stitching itself difficult. In such cases, there is concern regarding possible tendon damage. Many different suturing techniques are available for tendon repair. Rawson et al. described factors affecting repair success and highlight evolution and improvements in techniques and also how suture repairs might contribute to their own trauma [6].

Based on our clinical experience and impressions, we hypothesized that needle-shaped suture shuttles would require lower forces to pierce tendons and cause less tendon damage than suture retrievers. We were able to confirm this hypothesis in part. Combining all suture retrievers and shuttles, we found that lower forces were required to pierce a tendon when using a suture shuttle instead of a suture retriever. Unexpectedly, the overall lowest penetration forces were achieved using straight suture retrievers, though it must be noted that no truly straight suture shuttle was available. Among the angled instruments, however, suture shuttles required less penetration force and produced smaller tendon defects than retrievers. In addition, these angled suture shuttles showed the smallest lesion size (3 mm^2^), even smaller than the lesions from straight suture retrievers (5.1 mm^2^). Surprisingly, the instrument diameter does not seem to alter the required force.

To our knowledge, this is the first study systematically analyzing this topic. Chokshi *et al*. used different arthroscopic devices for suture passing and repair of the rotator cuff and tested these repairs to failure [7]. They conclude that larger holes created in the rotator cuff may compromise the integrity of the repair [7]. In 2003, Cummins and Murrell reported that the weak point of reconstruction is the tendon-suture interface rather than sutures, knots, or anchors in open rotator cuff repairs [8]. It has been shown experimentally that the highest pull out strength for sutures from the rotator cuff lies medial to the rotator cable [9], even though the tissue may also fail at that location, referred to as “medial cuff failure” [4, 5]. Explanatory factors such as potential overtensioning, and the effect of the braided suture materials on their passage through the rotator cuff, as well as holes created for reconstruction have been considered [10]. The last factor is our main interest in this study as often the tendon is pierced repeatedly until the ideal position for the suture is found. Intrasubstance tendon failures following successful suturing may be due to the considerably large holes created during stitching that contribute to weakening and tearing of the tendon. We are not aware of any in vivo studies that evaluate lesions made intraoperatively during repair using suture Retrievers and/or shuttles and the effect on the healing outcome of the repaired tendon.

We are aware of several limitations to this study: The number of suture retrievers and suture shuttles on the market is large and, unfortunately, not all manufacturers who were invited to provide instruments for testing participated. Furthermore, we addressed only instruments designed for either shuttling or retrieving of sutures. Other instruments, which allow for grasping tissue and needle passing the free end of the suture in one step, were not included. In addition, this biomechanical setup can, in the best case, only approximate the forces that occur intraoperatively during arthroscopic surgery, as soft-tissue tension, bony landmarks, and tissue behavior can only be partially reproduced in the laboratory setting. While the use of wax plates has the advantage of being highly standardized, it does not allow us to differentiate between cutting and merely displacing the pierced tissue, which may be mechanically important and will be addressed in future experiments. The displacement of the material also explains the larger diameter of the perforation marks in comparison to the size of the holes in the sieve while still avoiding contact. Nevertheless, this displacement also occurs in vivo as the instruments are applied.

Even though on average suture shuttles appeared to be more reliable for penetrating a tendon, it should be noted that retrievers can potentially reduce the number of operative steps, as they can also be used to manipulate sutures, are mechanically robust, can grasp and unload sutures inside the joint, and are equally useful for braided or monofilamentous suture materials. However, these advantages must be weighed against their potentially damaging effect on the tendon tissue, an effect that may be even larger when the tendon is not pierced perpendicularly to the tendon surface. More complex loading geometries will be considered and addressed in future biomechanical trials.

## Conclusions

Instruments designed and used for arthroscopic suture stitching were tested in an experimental setting. There were considerable differences between the tools regarding the force needed for tendon penetration and the size of the hole created. Angled suture retrievers need a higher force to perforate tendons and created larger lesions than suture shuttles did. These differences should be considered in the context of the additional features these tools offer, such as allowing the manipulation of sutures in the joint with the suture retriever. Even though the lesions created in the tendon are usually not visible during surgery, the possible damage created may be mechanically and biologically important.

## Additional file

Additional file 1: Table S1. Listing and illustration of all instruments used in respect to separation and subdivision. The mean values in N/mm and area in mm^2^ are shown of each instrument as well as of comparison of Suture Retrievers and Suture Shuttles and straight and angled instruments within each group. Values marked with identical symbol were compared and showed significant differences. Instruments, which showed a significantly less force or lesion size of all Instruments in comparison to the Arthrex™ Penetrator Suture Retriever II® 15° Up (bold) are marked for Force in N/mm (+) and for the area in mm^2^ in comparison to the Smith & Nephews™ ArthroPierce® 45° Right (bold). (PDF 566 kb)

## Abbreviations

ANOVA: Analysis of variance
IL: Illinois
mm: Millimeter
N: Newton
NJ: New Jersey
NY: New York
®: Registered Trade Mark
^TM^: Unregistered Trade Mark
USA: United States of America
USP: United States Pharmacopeia
°: Degrees
