# Supplementary material for: Arthroscopic suture retrievers and shuttles: a biomechanical investigation of the force required for tendon penetration and defect size
Source: BMC Musculoskelet Disord. 2015 Nov 17;16:357. doi: 10.1186/s12891-015-0794-9 (PMC4650195; doi:10.1186/s12891-015-0794-9)
Supplement: Additional file 1: Table S1. — Listing and illustration of all instruments used in respect to separation and subdivision. The mean values in N/mm and area in mm2 are shown of each instrument as well as of comparison of Suture Retrievers and Suture Shuttles and straight and angled instruments within each group. Values marked with identical symbol were compared and showed significant differences. Instruments, which showed a significantly less force or lesion size of all Instruments in comparison to the Arthrex™ Penetrator Suture Retriever II® 15° Up (bold) are marked for Force in N/mm (+) and for the area in mm2 in comparison to the Smith & Nephews™ ArthroPierce® 45° Right (bold). (PDF 566 kb) [file 12891_2015_794_MOESM1_ESM.pdf]

SUTURE RETRIEVER		FORCE (in N/mm)	LESION SIZE (in mm^2)	Force (N/mm) / *Lesion (mm^2)*, mean	Force (N/mm) / *Lesion (mm^2)*, mean
**Straight**					
Arthrex™ Penetrator Suture Retriever II® Straight	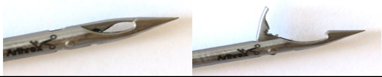	6^+	4.2^+	$9^†$ *5.1*	12 *4.9^**
Arthrex™ Rhino® Straight Tip	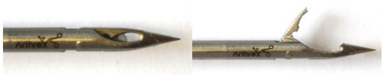	8^+	4.5^+		
Smith & Nephews™ Arthropierce® Straight	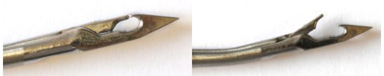	11	5.8		
Tornier™ Penetrating Grasper® Straight	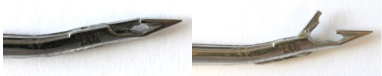	10	5.9		
**Angled**					
Arthrex™ Penetrator Suture Retriever II® 15° Up	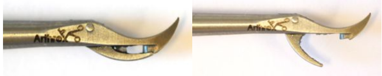	**19**	6.1	$13^†$ *4.9*	
Biomet™ Arthropasser® 35° Up	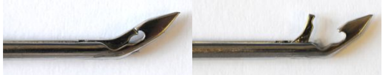	10	3.3^+		
Biomet™ Arthropasser® 45° Left	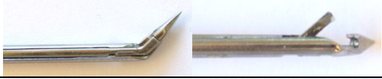	8^+	4.3^+		
Smith & Nephews™ ArthroPierce® 35° Up	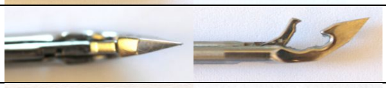	9	5.8		
Smith & Nephews™ ArthroPierce® 45° Right	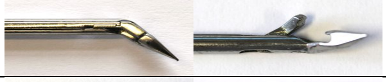	15	**7**		
Tornier™ Birdbeak® 35° Up	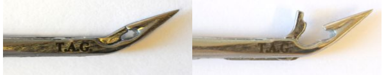	13	3^+		
Tornier™ Birdbeak® 45° Right	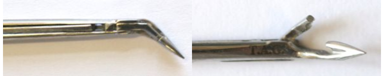	15	4.9		
Tornier™ CleverHook® Right	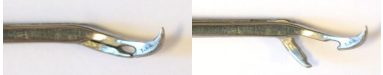	17	4.5^+		
**SUTURE SHUTTLE**					
**Straight**					
Arthrex™ Suture Lasso® SD Crescent	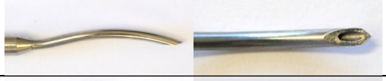	10	3.7^+	9 *3.3*	10 *3.1^**
Smith & Nephews™ Accu-Pass® Big Curve	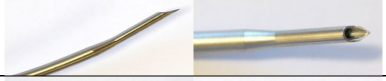	8^+	3.9^+		
Smith & Nephews™ Accu-Pass® Crescent	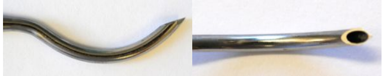	10	2.3^+		
**Angled**					
Arthrex™ Quick Pass Lasso® 90° Curve Straight	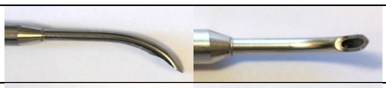	11	2^+	10 *3*	
Arthrex™ SutureLasso® SD 25° Tight Curve Left	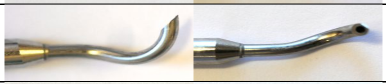	11	3.1^+		
ConMed Linvatec™ Spectrum® Suture Passer 45° Right	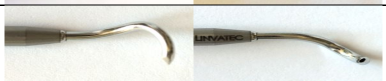	8^+	2.7^+		
ConMed Linvatec™ Spectrum® Suture Passer 60° Right	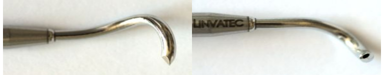	11	3^+		
Smith & Nephews™ Accu-Pass® Suture Shuttle 70°	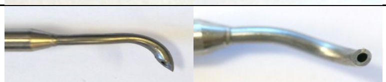	10	2.9^+		
Smith & Nephews™ Suture Shuttle® Left 45° Curve	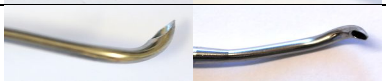	9	4.5^+		

Table 1: Listing and illustration of all instruments used in respect to separation and subdivision. The mean values in N/mm and area in mm^2 are shown of each instrument as well as of comparison of Suture Retrievers and Suture Shuttles and straight and angled instruments within each group. Values marked with identical symbol were compared and showed significant differences. Instruments, which showed a significantly less force or lesion size of all Instruments in comparison to the Arthrex™ Penetrator Suture Retriever II® 15° Up (bold) are marked for Force in N/mm (+) and for the area in mm^2 in comparison to the Smith & Nephews™ ArthroPierce® 45° Right (bold).
